# Cold and Spleen-Qi Deficiency Patterns in Korean Medicine Are Associated with Low Resting Metabolic Rate

**DOI:** 10.1155/2017/9532073

**Published:** 2017-03-06

**Authors:** Sujeong Mun, Sujung Kim, Kwang-Ho Bae, Siwoo Lee

**Affiliations:** Mibyeong Research Center, Korea Institute of Oriental Medicine, 1672 Yuseong-daero, Yuseong-gu, Daejeon 305-811, Republic of Korea

## Abstract

*Background.* Korean medicine (KM) patterns such as cold, heat, deficiency, and excess patterns have been associated with alterations of resting metabolic rate (RMR). However, the association of KM patterns with accurately measured body metabolic rate has not been investigated.* Methods.* Data on cold (CP), heat (HP), spleen-qi deficiency (SQDP), and kidney deficiency (KDP) patterns were extracted by a factor analysis of symptoms experienced by 954 participants. A multiple regression analysis was conducted to determine the association between KM patterns and RMR measured by an indirect calorimeter.* Results.* The CP and SQDP scores were higher and the HP score was lower in women. The HP and SQDP scores decreased with age, while KDP scores increased with age. A multiple regression analysis revealed that CP and SQDP scores were negatively associated with RMR independently of gender and age, and the CP remained significantly and negatively associated with RMR even after adjustment for fat-free mass.* Conclusions.* The underlying pathology of CP and SQDP might be associated with the body's metabolic rate. Further studies are needed to investigate the usefulness of RMR measurement in pattern identification and the association of CP and SQDP with metabolic disorders.

## 1. Introduction

Resting metabolic rate (RMR) is the amount of energy needed by the body to maintain homeostatic functions during resting conditions. It constitutes the largest fraction of total energy expenditure, accounting for about 65–70% [1]. RMR can be determined using indirect calorimetry by measuring oxygen consumption and carbon dioxide production, which is considered to be the gold standard for assessing RMR [2]. It has been demonstrated that RMR is influenced by various factors, including gender, age, ethnicity, and body composition. Among those, fat-free mass (FFM) has been recognized to be the major determinant of RMR [3]. Alterations in RMR are associated with obesity, metabolic syndrome, diabetes mellitus, multimorbidity, and even mortality [4–8].

Pattern identification, also known as syndrome differentiation, is an essential part of diagnosis in Korean medicine (KM). During the process of pattern identification, all symptoms and signs are analyzed to determine the patient's physical condition and the cause, nature, and location of a disease [9]. Pattern identification is principally used to guide medical intervention, and some studies have claimed that it improves the successful treatment outcome rate when used in both Eastern and Western interventions [10, 11].

Various books and research papers on KM have described the underlying pathology of KM patterns in relation to decreased or increased metabolic rate. For example, a cold pattern and a deficiency pattern are often reported to be related to a lowered body metabolism, while a heat pattern and excess pattern are related to excessive hyperactivity of the body metabolism, suggesting that the symptoms of the KM patterns such as cold/hot sensation in the body, decreased/increased sweating, and clear/red color of urine may be related to the altered body metabolic rate [12–15].

Few studies, however, have investigated the association of KM patterns with accurately measured body metabolic rate. To our knowledge, there was only one study conducted with twelve Japanese women that reported that RMR was lower in women with cold pattern than in women without cold pattern [16]. Thus, this study aims to further investigate the association of KM patterns with RMR and anthropometric and body composition measurements in a larger sample.

## 2. Materials and Methods

### 2.1. Participants

**Figure 1:**
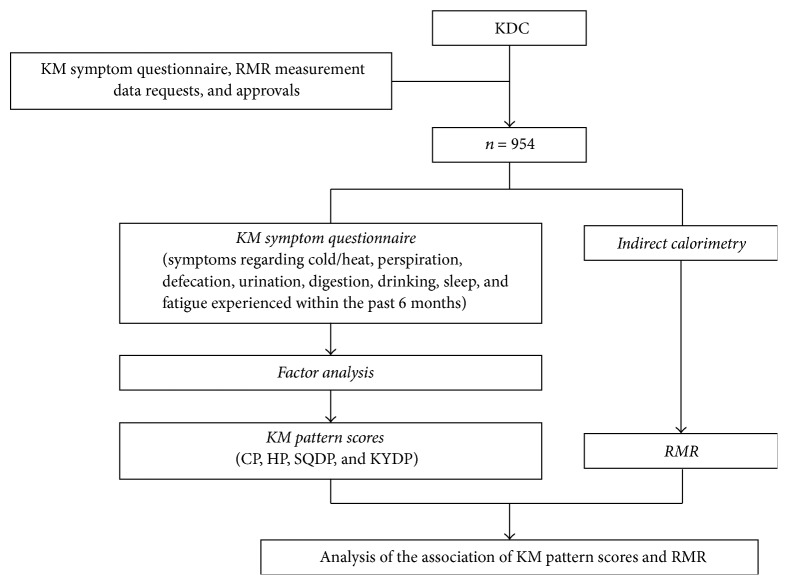
Flow chart of the study procedure. KDC, Korean Medicine Data Center; CP, cold pattern; SQDP, spleen-qi deficiency pattern; HP, heat pattern; KDP, kidney deficiency pattern.

This cross-sectional study was conducted between 2009 and 2015. The KM symptom questionnaire, RMR, and body composition measurement data were derived from the Korean Medicine Data Center (KDC) of the Korea Institute of Oriental Medicine [17] (Figure 1). A total of 954 healthy volunteers between 20 and 70 years of age without any chronic disease and history of hospitalization in the previous 5 years were included in the study. The study was approved by the Korea Institute of Oriental Medicine (Number I-1004/001-003) and informed consent was obtained from all participants prior to inclusion in the study.

### 2.2. Data Collection

#### 2.2.1. KM Patterns

Participants were asked to complete the KM symptom questionnaire that consisted of questions about symptoms experienced by the individual within the past 6 months. The symptoms referred to certain conditions, that is, cold/heat, perspiration, defecation, urination, digestion, drinking, sleep, and fatigue, which are deemed important during the basic examination in KM [12]. A factor analysis was conducted to identify symptom patterns. Four patterns, namely, cold pattern (CP), heat pattern (HP), spleen-qi deficiency pattern (SQDP), and kidney deficiency pattern (KDP), were extracted along with their respective scores (Supplementary Table S1 in Supplementary Material available online at https://doi.org/10.1155/2017/9532073). CP was characterized by cold sensation in the feet, cold sensation in the hands, no reddish urine, cold sensation in the abdomen, preference for drinking warm water, and frequent urination; HP was characterized by no aversion to cold, increased sweating, and no preference for drinking warm water; SQDP was characterized by irregular defecation, less frequent bowel movements, feeling of incomplete defecation, indigestion, and fatigue; and KDP was characterized by urination at night, awakening during the night, poor quality of sleep, frequent urination, and loose/watery stool (Supplementary Table S2).

#### 2.2.2. RMR Measurements

Participants were asked to fast and refrain from stimulants (smoking, alcohol, and coffee) and heavy exercise for at least 12 h prior to visiting the laboratory. RMR was measured with a breath-by-breath gas exchange analysis using an indirect calorimeter (Vmax ENCORE 29c; Sensormedic, Viasys HealthCare, Yorba Linda, CA, USA). The gas flow calibration as well as oxygen and carbon dioxide analyzer calibration was performed according to the manufacturer's instructions. RMR was measured for 20 min while participants were awake and lying in a supine position. Oxygen uptake and carbon dioxide production were measured and RMR was calculated using the Weir equation [18].

#### 2.2.3. Body Composition Measurements

Body height was measured with a digital scale (GL-150; G Tech International Co., Uijeongbu, Korea), and weight, fat-free mass (FFM), and body fat mass (BFM) were measured with a bioelectrical impedance analyzer (Inbody 720, InBody, Seoul, South Korea). Body mass index (BMI) was calculated as weight (kg) divided by the square of height (m).

### 2.3. Statistical Analysis

Sample characteristics were presented as the mean and standard deviation, and differences of sample characteristics and pattern scores between genders were compared using an independent *t*-test. The effect of age on pattern scores was analyzed by a simple regression within each gender. To examine the relationship of the pattern scores and anthropometric and body composition measurements, Pearson's correlation coefficients and partial correlation coefficients controlling for age and gender were estimated. To determine the association between pattern scores and RMR, a multiple regression analysis was conducted; this involved adjustment for age, gender, and FFM, which are known to be an influential factor of RMR [19]. A *p* value of less than 0.05 was considered statistically significant. Statistical analyses were performed using SPSS 22.0 (IBM, Chicago, IL, USA) and R (the R Foundation for Statistical Computing, Version 3.2.4).

## 3. Results

### 3.1. Characteristics of Participants

The characteristics of the study participants are shown in Table 1. Of the 954 participants, 500 (52.4%) were women and 454 (47.6%) were men. Their mean age was 37.8 ± 11.7 years. All anthropometric and body composition indices were statistically different between genders (*p* < 0.01). The mean RMR was higher in men (1579.5 ± 293.1 kcal/day) than in women (1197.2 ± 200.2 kcal/day) (*p* < 0.01).

### 3.2. Distribution of KM Pattern Scores by Gender and Age

**Figure 2:**
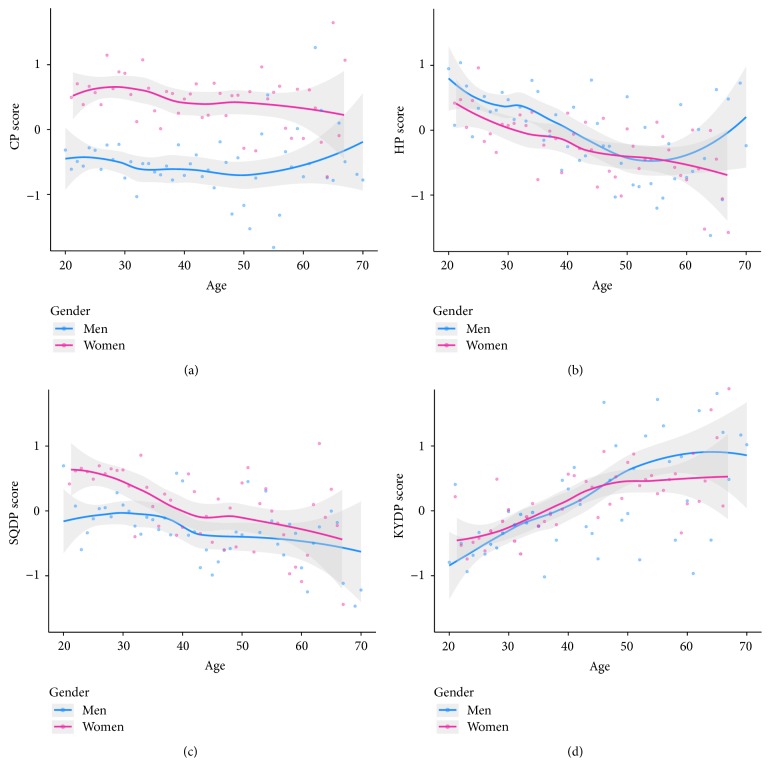
KM pattern scores by gender and age. Depicted are LOWESS smoothed lines and 95% confidence intervals (grey shade) with the observed means of pattern scores (dots) by gender and age. CP, cold pattern; SQDP, spleen-qi deficiency pattern; HP, heat pattern; KDP, kidney deficiency pattern.

The CP and SQDP scores were significantly higher (*p* < 0.01), while HP score was lower (*p* < 0.01) in women than in men. The CP score in women decreased with age (*p* < 0.05), which was not significant in men (*p* > 0.05). The SQDP and HP scores significantly decreased with age, while the KDP score increased with age in both genders (*p* < 0.05) (Figure 2).

### 3.3. Correlation of KM Pattern Scores with Anthropometric and Body Composition Indices

In correlation analysis with anthropometric and body composition indices, the CP and SQDP score showed negative correlations with height, weight, BMI, and FFM, while HP showed positive correlations with those indices. When adjusted for gender and age, the correlations of the CP, SQDP, and HP scores with anthropometric and body composition indices became weak but correlations with weight, BMI, and FFM remained significant. The KDP score has no significant correlation with any of the indices (Table 2).

### 3.4. Association of KM Pattern Scores with RMR

In Model 1, each of the KM pattern scores was entered into the regression model as an independent variable with RMR as a dependent variable. The CP and SQDP scores were negatively associated with RMR, while the HP score was positively associated with RMR. When gender and age were adjusted in Model 2, the CP and SQDP scores were significantly negatively associated with RMR. When additional adjustment of FFM was applied in Model 3, only the CP score was significantly associated with RMR (Table 3).

## 4. Discussion

The present study aimed to investigate the association between KM patterns and RMR. The pattern scores of CP, SQDP, HP, and KDP were different according to gender and age. In addition, CP, SQDP, and HP scores showed significant correlations with anthropometric and body composition indices. A multiple regression analysis revealed that CP and SQDP scores were negatively associated with RMR independently of gender and age, suggesting an association of the underlying pathology of CP and SQDP with altered RMR.

CP is one of the eight basic principles of pattern identification and believed to indicate the nature of imbalance in the body. CP is more common in women than in men [13, 20], and the same trend was observed in our results. The representative symptoms of CP, such as a cold sensation in the extremities and aversion to cold, are also more common in women [21–23]. This seems natural based on the report that the cutaneous hand blood flow of women at room temperature is lower than in men [24]. Also, when exposed to cold stimuli, the cutaneous blood flow is reduced more largely in women than in men [25].

According to the KM theory, CP encompasses symptoms of a cold sensation in the body, aversion to cold, lack of thirst, pale facial color, clear urine, and a desire to lie down; thus, its underlying pathology is often associated with decreased metabolism of the whole body [12–15]. A previous study reported that RMR is lower in women with CP than in women without CP; however, it included a very small sample size of twelve [16]. Our study included 954 participants, which is a comparatively larger sample than that of other studies using indirect calorimetry, to accurately estimate RMR. Our results were consistent with those of a previous study showing that the RMR was negatively associated with the CP score; this association remained significant after adjusting for gender, age, and even FFM, which is known to be the most influential determinant of RMR.

One of the possible explanations for the low RMR in individuals with CP could be their low thyroid function [16, 26], because altered level of thyroid hormone, even at subclinical level, has been known to be responsible for changes of thermogenesis, affecting energy expenditure and RMR [27]. Hypothyroidism could cause increased sensitivity to cold, which is a major symptom of CP. Indeed, hypothyroidism has been reported to be significantly more common in individuals diagnosed as CP than normal controls [13]. On the other hand, it has been also reported that differences of RMR, even when normalized by FFM, could be caused by differences in the composition of FFM, which can be accurately measured by using multiscan computerized axial tomography or magnetic resonance imaging [1, 28]. This is because FFM constitutes various kinds of tissues and organs that have different metabolic rates. Because our results indicate that the CP was significantly associated with RMR independently of the total FFM, the different composition of FFM in individuals with CP could be one of the explanations for the association between RMR and CP.

Several studies that investigated the different treatment effects in patients with rheumatoid arthritis found that effects of traditional East Asian medicine or even Western medicine could differ among patients with different characteristics related to the CP [11, 29]. This indicates that people with or without CP have different pathophysiological conditions that affect the treatment outcome. Because our results showed that RMR was significantly associated with CP, we could assume that one of the different pathological conditions between people with and without CP, which lead to different treatment effect, could be their different rate of body metabolism.

SQDP is mainly characterized by gastrointestinal problems, such as irregular defecation, reduced bowel movement, indigestion, and fatigue. This pattern is defined as a pathological change characterized by qi deficiency with impaired transporting and transforming function of the spleen [9]. Gastrointestinal problems could lead to altered eating habits that might reduce the total energy intake [30, 31]. RMR is known to decrease in response to restriction of energy intake to values below predictions based on body composition changes, possibly due to an adaptation which can limit weight loss and compromise the maintenance of a reduced body weight [32]. This mechanism could be one of the explanations for the low RMR in individuals with SQDP.

Our results suggest that CP and SQDP were significantly associated with RMR after adjustment for gender and age. This means that RMR is different according to the status of CP and SQDP in individuals with the same gender and age. Even though FFM is known to be the most influential determinant of RMR, FFM should be measured with a device such as a bioelectrical impedance analyzer or a dual-energy X-ray absorptiometer. Accordingly, the results indicate that evaluating CP and SQDP could be helpful to predict RMR of patients in a more practical way in clinical settings. Moreover, the CP was negatively associated with RMR after additional adjustment for FFM. Thus, consideration of the CP is recommended even when estimating an individual's RMR based on his/her FFM value.

The KM diagnostic process has been criticized for its subjectivity and dependence on the doctor's experience; thus the need to establish standardized diagnostic method using objective and quantitative methods has been asserted in the literature [33, 34]. Because our results showed the significant association of the CP and SQDP with RMR, we could assume that we may identify patients with CP and SQDP not only with traditionally used diagnostic method of questioning patients about symptoms and checking clinical signs such as tongue appearance and pulse evaluated by KM doctors, but also with objective and quantitative method of measuring RMR of patients. Also, our results suggest that reduced RMR could be one of the underlying pathologies of the relevant patterns, which means it is possible that the treatments which have been used for patients with the CP and SQDP are effective by means of directly or indirectly controlling the altered RMR of patients. Thus, the usefulness of measuring RMR in clinical practice for pattern identification and evaluation of clinical process should be further investigated in future studies.

Our results showed that the HP score was higher in men and showed positive associations with weight, BMI, FFM, and BFM and negative association with RMR, which was the opposite of CP. However, unlike the CP, the association of the HP with RMR was not significant after adjusting for gender and age. This is possibly due to the different composition of the symptom characteristics that are related to HP and CP in our study. The CP in our study was extracted by a factor analysis and mostly constituted symptoms related to thermal sensation of the body, while the HP mostly constituted symptoms related to preferred ambient temperature or sweating amount. However, according to KM theory, CP and HP are basically two patterns that reflect the nature of imbalance in the body and indicate the relative exuberance and debility of the yin and yang of the body. Consequently, the manifestations are conceptually contrast to each other; for example, the CP includes the symptoms of aversion to cold, preference for warmth, and a pale face, while the HP includes the symptoms of aversion to heat, preference for coolness, and a red face. Based on our results, it seems that the symptoms that are known to be relevant to the CP and HP are not necessarily analyzed as just one pattern. Our results could be interpreted as that the symptom pattern that was mostly about thermal sensation in the direction of cold was significantly associated with RMR and the symptom pattern that was mostly about preference for ambient temperature and sweating amount in the direction of heat were not significantly associated with RMR.

KDP is characterized by urination at night and sleep problems of awakening during the night and poor quality of sleep. The KDP score showed a significant increase with age, which is consistent with the KM theory that kidney function deteriorates with age [35]. The KDP score tended to decrease with increased RMR; however, this was not statistically significant.

Although there were studies that utilized a factor analysis of symptoms to statistically identify KM patterns [11, 29, 36], to the best of our knowledge, this is the first study to investigate the association of RMR with KM patterns based on a factor analysis. In pattern identification, symptoms have been utilized as key factors and the existence of a symptom pattern is screened rather than the existence of a single symptom. Predefined questionnaires for a specific pattern, which only include symptoms confined to that specific pattern, have been widely used in KM research, usually accompanying pattern scores of summing the number of pertinent symptoms [37–39]. However, in those studies, the statistically meaningful tendency of cooccurrence of symptoms could not be verified. In our study, a wide range of symptoms that are screened in basic examination in KM and possibly related to multiple KM patterns were analyzed by factor analysis, which yielded similar symptom patterns to CP, HP, SQDP, and KDP.

Some caution is needed when interpreting the results of this study. Firstly, data of healthy participants were analyzed in the study instead of patients with a specific disease. We expect symptoms related to KM patterns to be more prevalent and severe in patients with diseases and future studies to investigate the associations of KM patterns with RMR in various disease groups are required. Secondly, symptoms used in this study were self-reported, based on recall over the previous 6 months. Although self-administered questionnaires on symptoms have been widely used to evaluate an individual's health [40–42], this may be prone to a recall bias. We recommend that major clinical signs, such as the tongue appearance and pulse examination, should be included in future studies, to investigate their associations with symptom patterns and RMR.

## 5. Conclusions

Our results showed that the individuals with higher CP and SQDP scores have lower RMR when compared to individuals of the same gender and age with lower CP and SQDP scores, suggesting that the underlying pathology of CP and SQDP may be associated with the body's metabolic rate. In consideration of the effect of altered RMR on metabolic disorders, evaluating the CP and SQDP in patients with metabolic disorders is recommended. In addition, to complement the subjectivity of the current KM diagnosis process, further studies are required to investigate the usefulness of RMR measurement in CP and SQDP pattern identification.

## Supplementary Material

The Supplementary Material describes the detailed process of identifying symptom patterns, including the symptom questionnaire, the methods and results of factor analysis, and the comparison of factors extracted by factor analysis with KM patterns in the literature.

## Figures and Tables

**Table 1:** Characteristics of the study participants.

	All (*n* = 954)	Men (*n* = 454)	Women (*n* = 500)	*p* value
Height (cm)	166.0 ± 8.6	173.0 ± 5.8	159.7 ± 5.3	<0.001
Weight (kg)	64.0 ± 11.5	71.9 ± 10.0	56.9 ± 7.4	<0.001
BMI (kg/m^2^)	23.1 ± 3.0	24.0 ± 2.9	22.3 ± 2.9	<0.001
FFM (kg)	47.8 ± 9.9	56.7 ± 6.2	39.7 ± 3.9	<0.001
BFM (kg)	16.2 ± 5.7	15.2 ± 5.9	17.2 ± 5.3	<0.001
RMR (kcal/day)	1378.9 ± 313.5	1579.5 ± 293.1	1197.2 ± 200.2	<0.001

BMI, body mass index; FFM, fat-free mass; BFM, body fat mass; RMR, resting metabolic rate.

**Table 2:** Correlation coefficients of KM pattern scores with anthropometric and body composition indices.

	Unadjusted				Adjusted			
	CP	SQDP	HP	KDP	CP	SQDP	HP	KDP
Height	−0.291^**∗∗**^	−0.070^**∗**^	0.137^**∗∗**^	−0.112^**∗∗**^	0.035	−0.005	−0.005	−0.006
Weight	−0.439^**∗∗**^	−0.147^**∗∗**^	0.207^**∗∗**^	−0.038	−0.239^**∗∗**^	−0.086^**∗∗**^	0.168^**∗∗**^	0.009
BMI	−0.373^**∗∗**^	−0.140^**∗∗**^	0.171^**∗∗**^	0.050	−0.284^**∗∗**^	−0.083^**∗**^	0.186^**∗∗**^	0.023
FFM	−0.432^**∗∗**^	−0.148^**∗∗**^	0.158^**∗∗**^	−0.100^**∗∗**^	−0.168^**∗∗**^	−0.100^**∗∗**^	0.065^**∗**^	−0.037
BFM	−0.134^**∗∗**^	−0.042	0.142^**∗∗**^	0.099^**∗∗**^	−0.224^**∗∗**^	−0.044	0.206^**∗∗**^	0.048

Unadjusted, Pearson's correlation coefficients; adjusted, partial correlation coefficients adjusted for gender and age; CP, cold pattern; SQDP, spleen-qi deficiency pattern; HP, heat pattern; KDP, kidney deficiency pattern; ^*∗*^*p* < 0.05; ^*∗∗*^*p* < 0.01.

**Table 3:** Multiple regression analysis for the association of KM pattern scores and RMR.

	Model 1			Model 2			Model 3		
	*B*	SE	*p* value	*B*	SE	*p* value	*B*	SE	*p* value
CP	−89.9	7.8	**<0.001**	−33.4	7.2	**<0.001**	−15.6	6.5	**0.016**
SQDP	−18.5	7.8	**0.018**	−16.4	6.8	**0.016**	−9.4	6.0	0.119
HP	36.1	7.8	**<0.001**	12.2	6.8	0.075	3.5	6.0	0.564
KDP	−7.4	7.9	0.345	7.6	7.2	0.291	10.9	6.4	0.087

Model 1: unadjusted; Model 2: adjusted for gender and age; Model 3: adjusted for gender, age, and fat-free mass; *B*, unstandardized coefficients; SE, standard error of unstandardized coefficients; RMR, resting metabolic rate; CP, cold pattern; SQDP, spleen-qi deficiency pattern; HP, heat pattern; KDP, kidney deficiency pattern.
