# Supplementary material for: Cold and Spleen-Qi Deficiency Patterns in Korean Medicine Are Associated with Low Resting Metabolic Rate
Source: Evid Based Complement Alternat Med. 2017 Mar 6;2017:9532073. doi: 10.1155/2017/9532073 (PMC5358454; doi:10.1155/2017/9532073)
Supplement: Supplementary file 1 — The Supplementary Material describes the detailed process of identifying symptom patterns, including the symptom questionnaire, the methods and results of factor analysis, and the comparison of factors extracted by factor analysis with KM patterns in the literature. [file 9532073.f1.docx]

**Supplementary material**

**Factor analysis of symptoms to identify KM patterns and relevant scores**

1. **Symptom questionnaire**

Participants were asked to complete a questionnaire that consisted of questions about symptoms experienced by the individual within the past 6 months. The symptoms referred to certain conditions, i.e., cold/heat, perspiration, defecation, urination, digestion, drinking, sleep, and fatigue, which are deemed important during the basic examination in KM [1]. Accordingly, patients were asked about their usual condition in terms of the following: cold/heat sensation in the hands/feet/abdomen, aversion to cold/heat, sweating amount, the feeling after sweating, cold sweat, defecation frequency, feeling of incomplete defecation, stool type, urine color and turbidity, urination at night, urination frequency, indigestion, appetite, preferred temperature of drinking water, the amount drunk, sleep status, and fatigue.

1. **Factor analysis**

Responses to the questionnaire were composed of various types, including binary, Likert-type, and continuous data, and were all converted into binary data. Some pairs of items having a converse meaning tend to be highly correlated (e.g., frequent urination and less frequent urination, drinking warm water and drinking cold water); thus, one item in each pair was selected, based on clinical importance and magnitude of communalities [2]. In total, 25 symptoms were used in an exploratory factor analysis to identify symptom patterns by using tetrachoric correlations. The Kaiser−Meyer−Olkin measure of sampling adequacy and Bartlett’s test of sphericity were used to verify the appropriateness of performing a factor analysis. A scree plot was inspected to determine the number of factors; the eigenvalue of the retained factors needed to exceed 1. We assumed that factors might be associated with each other; thus, we used an oblique (oblimin) rotation of factor loading matrices. Items with factor loading > |0.30| were deemed significant. Factor scores were computed using regression for each individual to indicate the extent to which the individual related to that factor. Factor analysis was performed using R (The R Foundation for Statistical Computing, Version 3.2.4) with its libraries of psych [3], and GPArotation [4].

1. **Results**

The Kaiser−Meyer−Olkin measure of sampling adequacy was 0.65 and Bartlett’s test of sphericity was significant (*p* < 0.001), indicating that the data were appropriate for factor analysis. The number of factors with eigenvalues >1 was four, and a change in the curvature in the scree plot was also observed after the fourth factor.

The four factors (symptom patterns) accounted for 45.3% of variations in all the items. Factor 1 (eigenvalue 3.059) included a cold sensation in the feet, cold sensation in the hands, cold sensation in the abdomen, and indigestion. Reddish urine was negatively loaded on this factor (factor loading -0.619). Factor 2 (eigenvalue 2.978) included irregular defecation, fewer bowel movements, cloudy urine, a feeling of incomplete defecation, and difficulty falling asleep. Indigestion was also loaded on this factor (factor loading 0.411), as well as on the first factor (factor loading 0.430). Factor 3 (eigenvalue 2.725) included aversion to both cold and heat, increased sweating, fatigue after sweating, and cold sweat. Aversion to cold was negatively loaded on this factor (factor loading -0.839). Factor 4 (eigenvalue 2.574) included urination at night, awakening during the night, and poor quality of sleep; reddish urine was also loaded on this factor (factor loading 0.502). (Table S1).

We compared the extracted factors with relevant KM patterns in the literature, including the WHO international standard terminology of traditional medicine [5] and relevant publications to each corresponding pattern (Table S2). According to the result, factors 1 through 4 were interpreted as cold pattern (CP), spleen-qi deficiency pattern (SQDP), heat pattern (HP), and kidney deficiency pattern (KDP), respectively.

**Table S1**: Factor loadings for the four factors (symptom patterns) identified by factor analysis.

|  | **Factor 1** | **Factor 2** | **Factor 3** | **Factor 4** |
| --- | --- | --- | --- | --- |
| Cold sensation in the feet | **0.869** | 0.027 | -0.091 | 0.036 |
| Cold sensation in the hands | **0.798** | 0.064 | -0.142 | 0.020 |
| Reddish urine | **-0.619** | 0.198 | -0.227 | **0.502** |
| Cold sensation in the abdomen | **0.572** | 0.133 | 0.058 | 0.223 |
| Indigestion | **0.430** | **0.411** | 0.126 | -0.005 |
| Preference for drinking warm water | **0.381** | -0.199 | **-0.333** | 0.263 |
| Decreased appetite | **0.330** | 0.242 | 0.211 | 0.138 |
| Irregular defecation | -0.006 | **0.801** | -0.024 | -0.046 |
| Less frequent bowel movements | 0.084 | **0.757** | -0.181 | -0.120 |
| Cloudy urine | -0.216 | **0.549** | -0.162 | -0.001 |
| Feeling of incomplete defecation | 0.116 | **0.494** | 0.222 | 0.199 |
| Difficulty falling asleep | 0.032 | **0.428** | **0.333** | **0.314** |
| Fatigue | 0.050 | **0.396** | 0.212 | 0.226 |
| Drink less water | 0.165 | **0.329** | -0.079 | -0.209 |
| Aversion to cold | 0.144 | 0.090 | **-0.839** | 0.098 |
| Aversion to both cold and heat | 0.150 | 0.030 | **0.757** | 0.039 |
| Increased sweating | -0.228 | -0.193 | **0.616** | -0.042 |
| Fatigue after sweating | **0.305** | 0.158 | **0.451** | 0.070 |
| Cold sweat | -0.177 | 0.018 | **0.427** | **0.317** |
| Urination at night | -0.034 | -0.283 | -0.168 | **0.725** |
| Awakening during the night | 0.092 | -0.008 | 0.002 | **0.719** |
| Poor quality of sleep | 0.066 | 0.294 | 0.248 | **0.601** |
| Weak urine stream | 0.162 | -0.081 | -0.036 | **0.407** |
| Frequent urination | **0.316** | -0.300 | 0.038 | **0.355** |
| Loose/watery stool | -0.081 | 0.144 | 0.216 | **0.309** |
| **Eigenvalue** | 3.059 | 2.978 | 2.725 | 2.574 |
| **Proportion of variance explained (%)** | 12.2 | 11.9 | 10.9 | 10.3 |
| **KM pattern** | CP | SQDP | HP | KDP |

CP, cold pattern; SQDP, spleen-qi deficiency pattern; HP, heat pattern; KDP, kidney deficiency pattern

**Table S2:** Comparison of factors (symptom patterns) extracted by factor analysis with KM patterns in the literature.

| **Factors extracted by factor analysis** | | **KM patterns in the literature** | |
| --- | --- | --- | --- |
| **Factor** | **Symptoms** | **KM pattern** | **Symptoms** |
| Factor 1 | **Cold sensation in the feet**  **Cold sensation in the hands**  **No reddish urine^*^**  **Cold sensation in the abdomen**  Indigestion  **Preference for drinking warm water**  Decreased appetite  **Frequent urination**  Fatigue after sweating | CP | Cold limbs, cold abdomen, drinking warm water, long voidings of clear urine, liking for hot drinks, aversion to cold, cold pain with preference for heat, absence of thirst, thin clear sputum and nasal mucus, and loose bowels [1,5,6] |
| Factor 2 | **Irregular defecation**  **Less frequent bowel movements**  Cloudy urine  **Feeling of incomplete defecation**  Difficulty falling asleep  **Indigestion**  **Fatigue**  Drinking less water | SQDP | Altered bowel habits, indigestion, fatigue, lassitude, feeling of incomplete bowel movement, abdominal distension, dizziness, anorexia, loose stools, symptom exacerbation, especially postprandially, and food sensitivities [1,5,7] |
| Factor 3 | **No aversion to cold^*^**  Aversion to both cold and heat  **Increased sweating**  Fatigue after sweating  Cold sweat  Difficulty falling asleep  **No preference for drinking warm water^*^** | HP | Aversion to heat, sweating, drinking cold water, preference for cold/warm body, hot/burning sensation in body, hot breath, thirst, irritability and vexation, thick yellow sputum and nasal mucus, short voidings of dark-colored urine, constipation, and bitter taste in the mouth [1,5,6] |
| Factor 4 | **Urination at night**  **Awakening during the night**  **Poor quality of sleep**  Reddish urine  Weak urine stream  **Frequent urination**  Difficulty falling asleep  **Loose/watery stool** | KDP | Frequent urination, urination at night, easily waking and restless sleep, dizziness, forgetfulness, tinnitus, backache, lack of libido, lassitude, incontinence in the elderly, slight dribbling, loose stools, depression, and impotence [1,5,8] |

Symptoms of each factor that accord with the description of the corresponding KM patterns in the literature were indicated in bold; ^*^, Symptoms with negative factor loading were expressed as non-existence of the symptom; CP, cold pattern; SQDP, spleen-qi deficiency pattern; HP, heat pattern; KDP, kidney deficiency pattern

**References**

1. Deng T. Practical Diagnosis in Traditional Chinese Medicine. Edinburgh: Churchill Livingstone; 1999.

2. Tanaka Y, Kodake K. A method of variable selection in factor analysis and its numerical investigation. Behaviormetrika. 1981;8:49–61.

3. Revelle W. psych: Procedures for Personality and Psychological Research (version 1.5.8) [Internet]. 2015 [cited 2016 Jul 21]. Available from: http://cran.r-project.org/package=psych

4. Bernaards CA. Gradient Projection Algorithms and Software for Arbitrary Rotation Criteria in Factor Analysis. Educ. Psychol. Meas. 2005;65:676–96.

5. WHO Regional Office for the Western Pacific. WHO International Standard Terminologies on Traditional Medicine in the Western Pacific Region. Geneva: World Health Organization; 2007.

6. Yeo M, Park K, Bae K, Jang E, Lee Y. Development on the questionnaire of cold-heat pattern identification based on usual symptoms for health promotion focused on reliability study. J. Physiol. Pathol. Korean Med. 2016;30.

7. Tan S, Tillisch K, Bolus SR, Olivas TI, Spiegel BMR, Naliboff B, et al. Traditional Chinese medicine based subgrouping of irritable bowel syndrome patients. Am. J. Chin. Med. 2005;33:365–79.

8. Butler BL. The Treatment of Urinary Incontinence and Frequent Urination : How Modern Scientific Research Can Inform. J. Chinese Med. 2011;52–7.
